# An automated and reliable method for breath detection during variable mask pressures in awake and sleeping humans

**DOI:** 10.1371/journal.pone.0179030

**Published:** 2017-06-13

**Authors:** Chinh D. Nguyen, Jason Amatoury, Jayne C. Carberry, Danny J. Eckert

**Affiliations:** 1 Neuroscience Research Australia (NeuRA), Randwick, New South Wales, Australia; 2 Woolcock Institute of Medical Research and Sydney Medical School, University of Sydney, Glebe, New South Wales, Australia; 3 School of Medical Sciences, University of New South Wales, Sydney, New South Wales, Australia; University of Adelaide, AUSTRALIA

## Abstract

Accurate breath detection is crucial in sleep and respiratory physiology research and in several clinical settings. However, this process is technically challenging due to measurement and physiological artifacts and other factors such as variable leaks in the breathing circuit. Recently developed techniques to quantify the multiple causes of obstructive sleep apnea, require intermittent changes in airway pressure applied to a breathing mask. This presents an additional unique challenge for breath detection. Traditional algorithms often require drift correction. However, this is an empirical operation potentially prone to human error. This paper presents a new algorithm for breath detection during variable mask pressures in awake and sleeping humans based on physiological landmarks detected in the airflow or epiglottic pressure signal (Pepi). The algorithms were validated using simulated data from a mathematical model and against the standard visual detection approach in 4 healthy individuals and 6 patients with sleep apnea during variable mask pressure conditions. Using the flow signal, the algorithm correctly identified 97.6% of breaths with a mean difference±SD in the onsets of respiratory phase compared to expert visual detection of 23±89ms for inspiration and 6±56ms for expiration during wakefulness and 10±74ms for inspiration and 3±28 ms for expiration with variable mask pressures during sleep. Using the Pepi signal, the algorithm correctly identified 89% of the breaths with accuracy of 31±156ms for inspiration and 9±147ms for expiration compared to expert visual detection during variable mask pressures asleep. The algorithm had excellent performance in response to baseline drifts and noise during variable mask pressure conditions. This new algorithm can be used for accurate breath detection including during variable mask pressure conditions which represents a major advance over existing time-consuming manual approaches.

## Introduction

Accurate breath detection is crucial in sleep and respiratory physiology research and in clinical practice. Indeed, in order to quantify key breathing variables such as minute ventilation and peak inspiratory airflow, accurate identification of inspiration and expiration is required. Breath detection also has multiple applied and cross-disciplinary applications. For instance, breath detection is required to characterize interactions between the respiratory system and other organs such as the heart (e.g. respiratory sinus arrhythmia [1] and cardiorespiratory synchronization [1–4]), and neurological function [5, 6].

Despite its importance, breath detection is technically challenging. Physiological events such as sighs, swallows, transient reductions and pauses (hypopneas and apneas) in breathing during sleep recordings, as well as measurement artifacts including signal drift, EKG artifact, electrical noise on the airflow signal and mask leaks, each present unique challenges when attempting to quantify breath timing accurately. Manual detection and calculation of respiratory parameters are time-consuming and potentially prone to human error and impractical for large data sets. Accordingly, several algorithms have been developed for automated breath detection [7–9]. Most use an airflow signal, a volume signal, or both, and apply different threshold criteria to identify breaths [7–9]. However, measurement artifacts, especially baseline volume shifts, can render these algorithms inaccurate [7, 8]. Drift correction is often used to counteract this problem. However, this is an empirical operation and is prone to error.

Recent advances in the pathophysiology of obstructive sleep apnea (OSA), a common sleep-related breathing disorder characterized by repetitive narrowing and closure of the upper airway during sleep, indicate that there are at least four key causes [10, 11]. These include upper airway anatomy/collapsibility, the respiratory arousal threshold, pharyngeal dilator muscle responsiveness and respiratory control instability [10]. Quantification of each parameter relies heavily on accurate detection of respiratory phase. The gold standard approach to measure these causes or “phenotypic traits” requires transient changes in mask pressure (positive and negative suction pressure) to induce varying degrees of upper airway collapse [10–18]. However, automated breath detection is particularly challenging during these conditions due to frequent and large volume drifts that accompany the transient changes in mask pressure and noise artifact from the pressure generation device. There are limited effective computational tools available to deal with this problem. Thus, breath detection under these conditions remains largely manual, labor intensive and is potentially prone to error. Accordingly, to facilitate translation of these and related applications, development of accurate, automated tools for breath detection to address these barriers is required.

Thus, the aim of this study was to develop an algorithm to accurately detect the onset of inspiration and expiration in humans during variable mask pressure conditions and test its accuracy using multiple approaches.

## Materials and methods

### Traditional approach and methodology for breath detection algorithms

**Fig 1:**
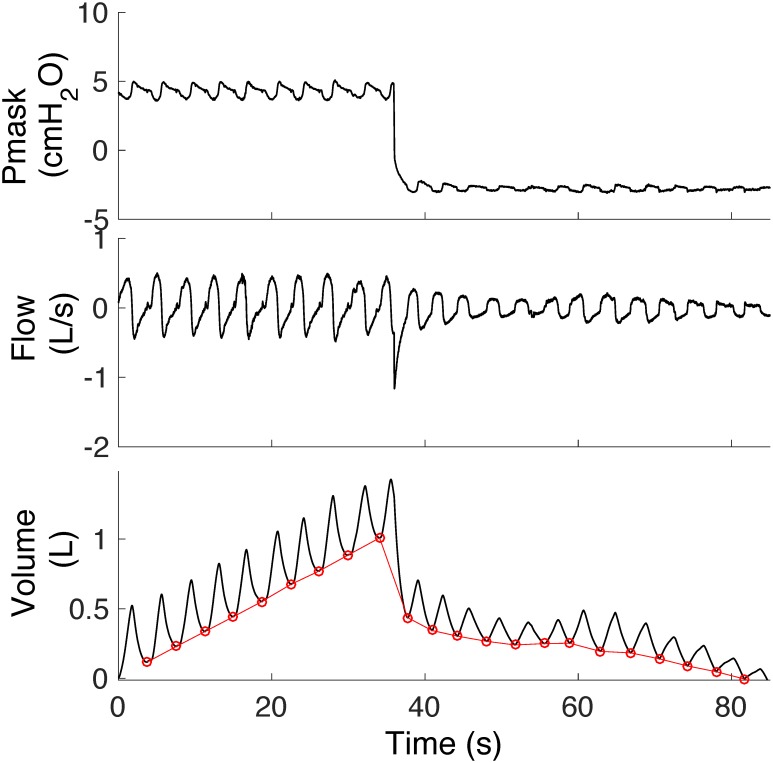
Volume drift during a transient reduction in continuous positive airway pressure delivered via a nasal mask (Pmask). End of expiratory volume signal (red circles) often does not follow a linear upward/downward direction during this period. Volume drift correction is an empirical process potentially prone to human error.

Traditional breath detection algorithms define the onsets of inspiration and expiration as the points at which the drift-corrected volume signal attains its minimum and maximum values, or where the flow signal crosses zero [7–9]. The common signal processing tasks include: 1) integration of the flow signal to obtain volume using a numerical integration algorithm, 2) volume drift correction, often performed by subtracting the straight line fitted to the end-expiratory points from the volume signal, and 3) minima and maxima of the drift-corrected volume signal (possible onsets of inspiration and expiration, respectively) (see [8] for further detail). However, the volume drift correction is an empirical process and becomes problematic when the drift does not increase or decrease linearly. Thus, this approach is inaccurate during conditions where mask pressure is variable or breathing is unstable (Fig 1).

The zero-crossing algorithm approach to identify the onsets of inspiration and expiration also has several limitations. This algorithm requires two conditions to be satisfied: 1) no significant zero offset in the flow signal, and 2) no oscillations around baseline flow as any flow crossings including even small deviations unrelated to breathing will be incorrectly detected [8]. Both of these conditions are violated when there is a strong drift in the flow baseline which occurs as a consequence of air leaks and unstable breathing.

### Novel breath detection algorithm

#### Physiological landmark of inspiratory and expiratory onsets

**Fig 2:**
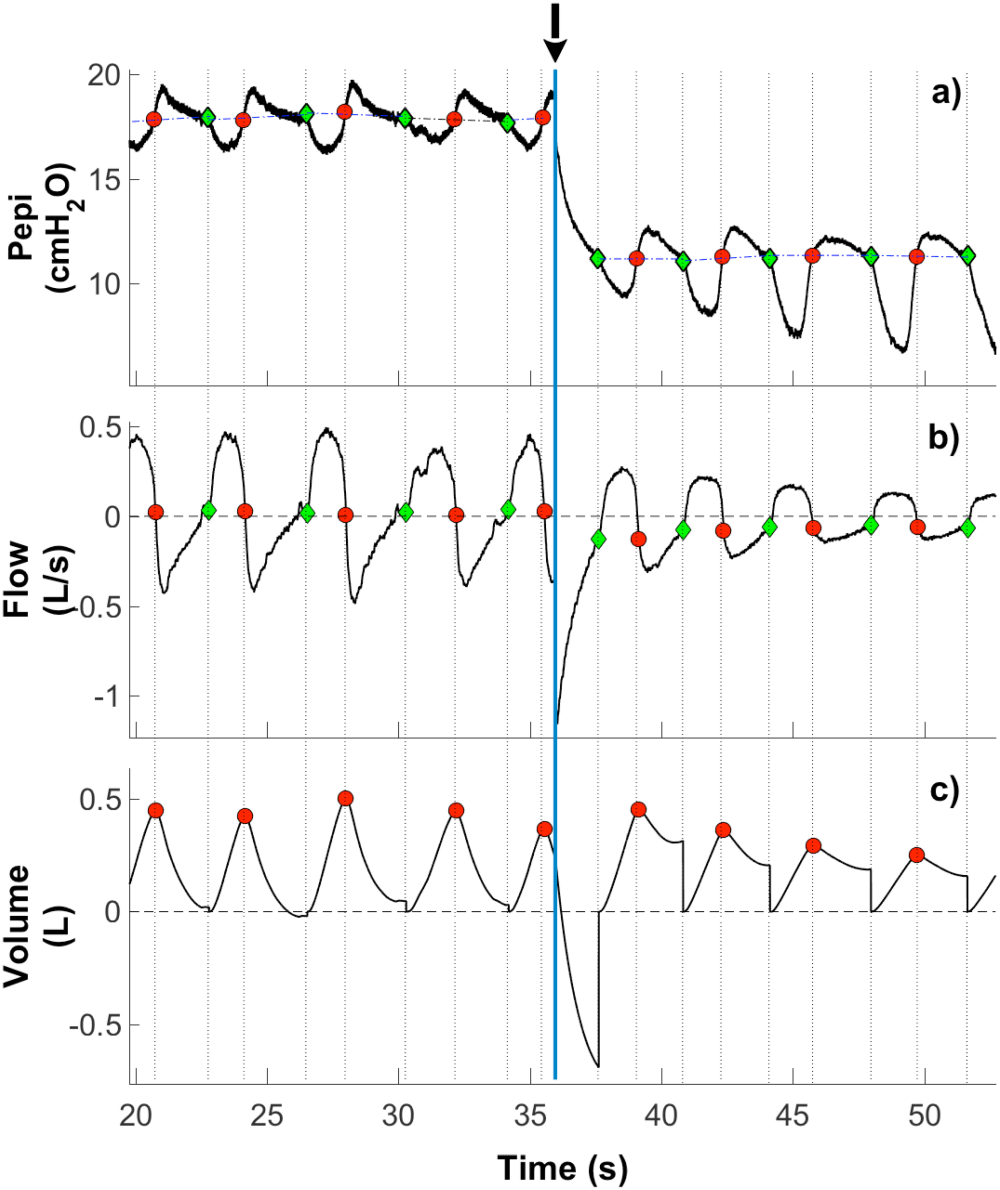
Physiological landmarks of true onsets of inspiration as inflection points (green diamonds) in flow (a) and epiglottic pressure (Pepi) signals (b). Note the alignment of the inflection points in the flow and Pepi signals whereby Pepi sharply decreases at the onset of inspiration. When the flow signal (b) is used, the onsets of expiration (red circles) are defined as the points at which the algorithm corrected integrated volume signal (c) attains its maximum value between two adjacent inspiratory onsets. When the Pepi signal (a) is used, the expiratory onsets (red circles) are defined as the interception points between the straight lines (connecting the inspiratory onsets of two consecutive breaths, dash-dot lines) and Pepi waveform. The black arrow and blue vertical line indicate the start of a transient reduction in continuous positive airway pressure (CPAP).

Our proposed algorithm defines the “true” onset of inspiration as the points at which inspiratory effort commences. This can be identified as an inflection point in the flow or epiglottic pressure (Pepi) signals (Pepi, measured by an epiglottic catheter). Fig 2 shows that the inflection point in flow aligns with the inflection point in the Pepi signal. Specifically, Pepi sharply decreases at the onset of inspiration. The inflection point in Pepi also provides important physiological information analogous to “breath timing” during situations where airflow is absent (e.g. during obstructive apneas). Detection of the onset of inspiratory effort using airway pressure transducers such as Pepi is also useful for estimating intrathoracic pressure gradients and for correcting baseline drift in these signals. Accordingly, our proposed algorithm focuses on reliable detection of inflection points in flow and Pepi signals. Once these components are clearly defined it is then possible to calculate key respiratory (e.g. tidal volume, minute ventilation, peak flow) and related variables that rely on accurate breath detection (e.g. airway resistance, pharyngeal pressure swings, inspiratory and expiratory muscle activity). Accurate quantification of these parameters is often crucial for sleep and respiratory research [19–22].

When using the flow signal, the onset of expiration is defined as the point at which the algorithm corrected integrated volume signal (generated by integrating flow signal value between two adjacent inspiratory onsets) attains its maximum (Fig 2b and 2c). However, it is more challenging to identify expiratory onset using the Pepi signal alone as unlike inspiration there is no clear marker on this signal that could be used to detect expiratory onsets. In this study, expiratory onset using the Pepi signal was defined as the point of intercept between a straight line (connecting the inspiratory onsets of two consecutive breaths) and the Pepi waveform (Fig 2a).

#### Automated detection of the inflection points with noisy flow and pressure signals

**Fig 3:**
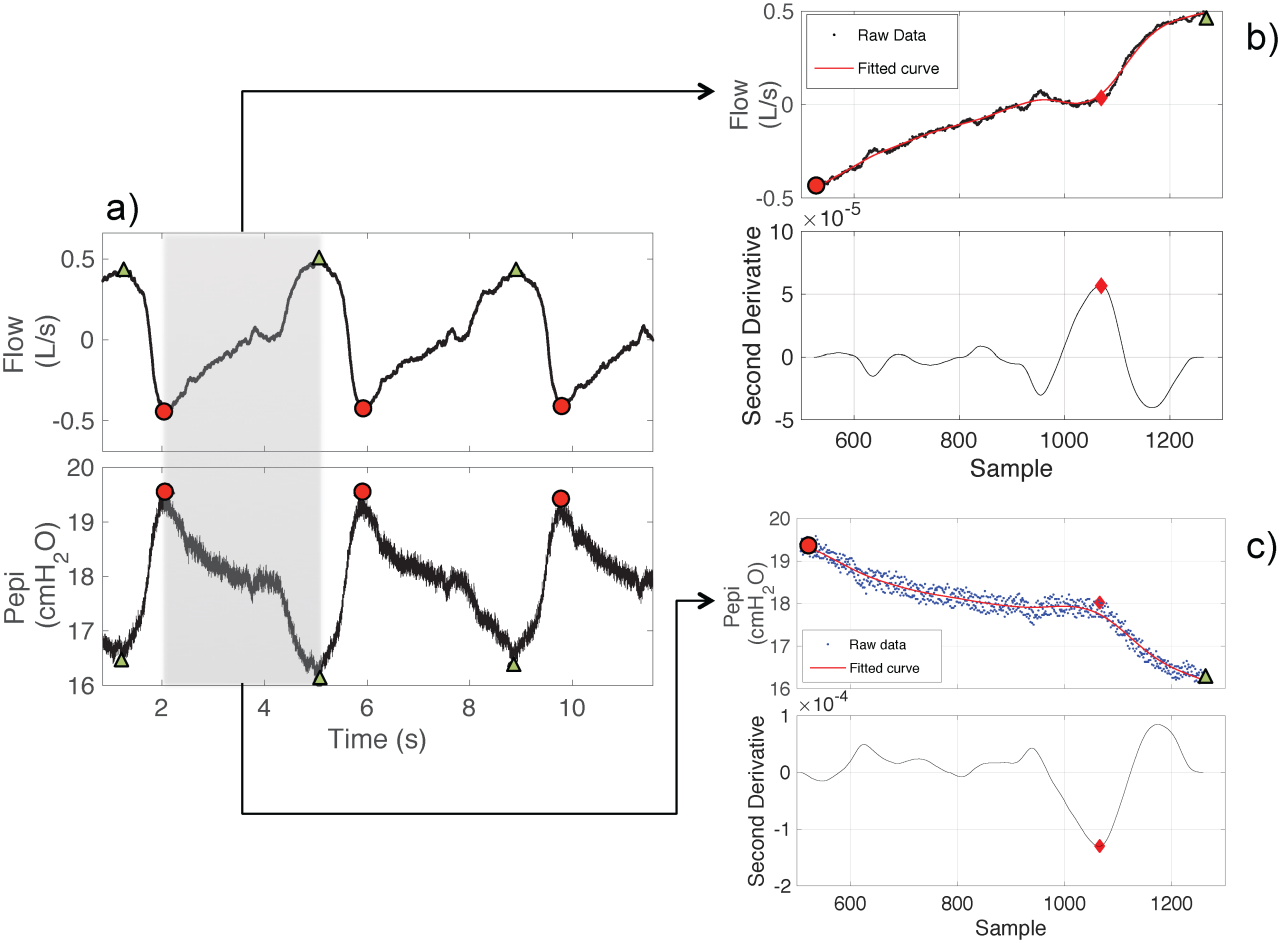
Key steps of the proposed breath detection algorithm. Briefly, peaks and valleys of flow (or pressure) are identified (circles and triangular points) (a), smoothing curves are fitted to raw data (b-c, upper panels) and the second derivatives of the fitted curves are calculated (b-c, lower panels). Onsets of inspiration are located at the maximum and minimum points (diamonds) of the second derivate of flow and pressure signal, respectively (b-c, lower panels). Pepi: epiglottic pressure.

The inflection point of the flow signal is detected as the point where its second derivative reaches its maximum value (reflecting the peak acceleration of changes in the shape of flow signal). There are three main steps required to apply this concept to detect inflection points in noisy flow and pressure signals (Fig 3). These include: 1) detection of signal peaks and valleys followed by selection of each segment of the signal connecting the corresponding peak, valley and possible inflection point (Fig 3a). A low pass filter with a cut-off frequency of 2 Hz was applied to the signal and peak detection was performed on the filtered signal using the MATLAB function ‘findpeaks’ with a threshold for breath duration of one second, 2) fitting a smoothing spline with a smoothing parameter (*SP)* through the segment (Fig 3b, upper panel), SP was set to 0.95, and 3) calculation of the second derivative of the fitted smoothing spline to find the location where it attains the maximum value (Fig 3b, lower panel). Similarly, the inflection point in the pressure signal is detected where its second derivative attains its minimum value (Fig 3c).

### Datasets for validation

#### Synthetic dataset from a mathematical model

**Fig 4:**
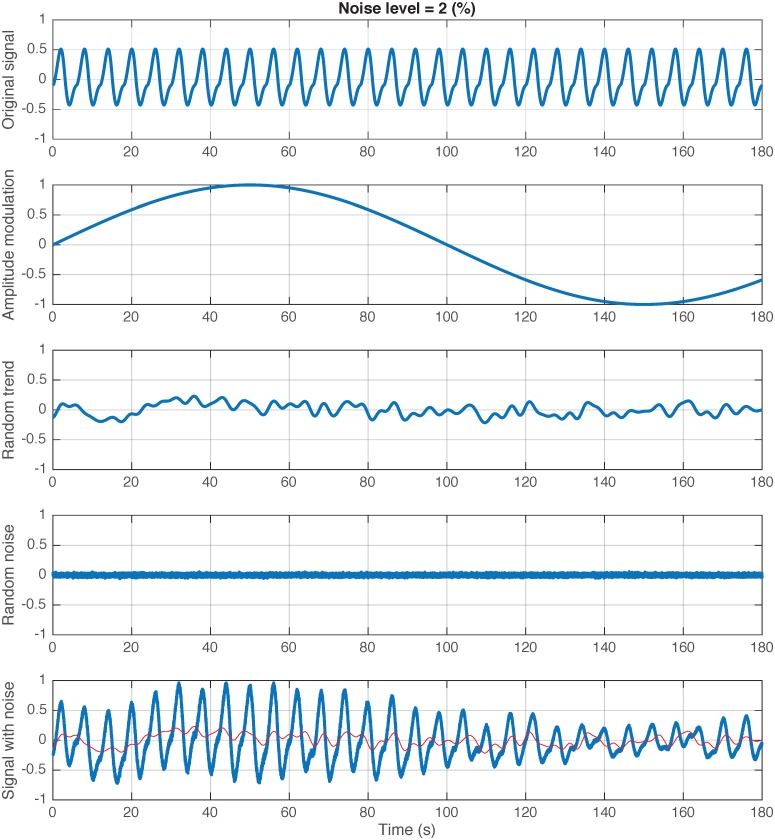
Mathematical model of a flow signal that contains inflection points. Amplitude modulation, random trend and random noise were added to the model to simulate fluctuations in amplitude, baseline shift and noise, respectively. For further detail on the model refer to the text and Figure A in S1 File. Note: the red line shows the random trend simulating fluctuations in baseline shift of the flow signal, which consequently renders traditional volume-drift correction algorithms inaccurate. Noise level is set to 2% in this example.

We created a mathematical model to validate our algorithm against different levels of noise and fluctuation in the flow amplitude and baseline, and across a range of sampling frequencies. The model was comprised of combined sine and cosine waveforms with known inflection points, which were set by a square waveform. Amplitude modulation, random trend and random noise were then added to the model to simulate fluctuations in amplitude of flow, fluctuations in flow baseline and signal noise, respectively (Fig 4 and see Figure A in S1 File for further details).

The signal with inflection points was generated according to the following model:

$$X\left(t\right)=\;Y\left(t\right)[A_{1}\text{sin}(2\pi f_{1}t/f_{s}+\varphi )]+\;A_{2}\text{cos}(2\pi f_{1}t/f_{s}+\varphi )$$ (1)

where *A*_1_ = 0.25, *A*_2_ = 0.1, *f*_1_ is breathing frequency, *f*_*s*_ is sampling frequency, *Y*(*t*) is a square wave function with duty cycle = 50% and *φ* is an initial phase of the signal (rad), *φ* ∈ [0,2*π*].

Then random noise, amplitude modulation and random trend were added to the model:

$$X_{noisy}\left(t\right)=\left[X\left(t\right)+\xi_{n}\right]\left[1+A_{m}\text{sin}(2\pi f_{m}t/f_{s}+\varphi \right)]\;+\xi_{trend}]$$ (2)

where *ξ*_*n*_ is Gaussian white noise with zero mean and standard deviation = noise level x square root of amplitude of X(t); *A*_*m*_ and *f*_*m*_ are amplitude and frequency of the amplitude modulating signal (*A*_*m*_ = 0.5,*f*_*m*_ = 0.005); *ξ*_*trend*_ is fractal Gaussian noise with zero mean, standard deviation = 0.1 and Hurst exponent = 0.9, generated using Davies and Harte’s algorithm [23]. The amplitude and frequency modulation were used to simulate fluctuations in the amplitude and frequency of the flow signals. Whereas, the fractal Gaussian noise with Hurst exponent = 0.9 was used to simulate the breath-by-breath fluctuation in flow baseline.

This model was used to generate a synthetic dataset as follows: 1) with each breathing frequency (from 8 to 17 breath/min), a segment of 100 breaths was generated, and the signal of a total of 1000 breaths was formed by concatenating these segments to cover a spectrum of the normal breathing frequency, 2) the process was repeated with each combination of sampling frequency {fs = [50, 100, 250, 500 and 1000] Hz} and noise level {noiselevel = [0, 2, 5, 7 and 10] (%)}, to generate a dataset of 1000 breath signals with different breath frequency, sampling frequency and noise levels (Fig 4).

#### Clinical dataset

A dataset derived from 6 obstructive sleep apnea (OSA) patients and 4 healthy controls was used to validate the breath detection algorithm. Participants were fitted with a CPAP mask (Gel Mask; Philips Respironics, Murrysville, PA) attached to a pneumotachograph (model 3700A; Hans Rudolf Inc., Kansas City, MO) and differential pressure transducers (Validyne Corporation, Northbridge, CA) to measure airflow and mask pressure. An epiglottic pressure (Pepi) catheter (model MCP-500; Millar, Houston, TX) was placed 1 to 2 cm below the base of tongue. The epiglottic pressure sensor was taped to the nostril and passed through a port in the CPAP mask. Flow was sampled at 250 Hz, and mask and epiglottic pressures were sampled at 1000 Hz.

Participants were studied under several conditions, including: awake quiet breathing, during stable non-rapid eye movement (NREM) sleep on therapeutic continuous positive airway pressure (CPAP), and during transient reductions in CPAP to cause varying levels of airflow limitation. Periods of wakefulness and NREM sleep were confirmed by electroencephalography (EEG). EEG, electrooculograms, and surface submentalis electromyograms were used for sleep staging and scoring arousals according to standard criteria by an experienced sleep technician. The study was approved by the University of New South Wales Human Research Ethics Committee and all participants provided informed written consent to participate in the protocol. In each participant, 2 minutes of wakefulness data, 1 minute of sleep data on therapeutic CPAP (or 4–5cmH_2_O in the healthy controls) immediately preceding each CPAP reduction and data from 4 transient CPAP reductions also during NREM sleep, were extracted for manual breath detection analysis. Two of the CPAP reductions were selected at random to yield mild-moderate airflow limitation where peak inspiratory flow (PIF) for all of the breaths were greater than 0.2 (L/s) during the reduction in CPAP. The remaining two CPAP reductions were randomly selected to yield low flow, where there were one or more breaths with PIF ≤ 0.2 (L/s) lasting greater than 10 sec. Manual breath detection was performed by placing cursors at the onset of inspiration and expiration by an experienced investigator blinded to the algorithm results using SPIKE2 software (Cambridge Electronic Design, UK).

### Comparison process and statistical analyses

We have implemented our novel algorithm in a custom signal-processing module, developed in MATLAB (version 8, The MathWorks, Natick, MA, USA). We applied our software to detect inspiratory and expiratory onsets and compared the results against the true values (set by square waves) in the synthetic dataset and the expert’s visual detection in the clinical dataset. Within the clinical dataset, flow signals were used for breath detection during wakefulness, therapeutic CPAP and mild-moderate airflow limitation conditions. Conversely, epiglottic pressure signals were used for breath detection during low flow conditions. Key respiratory parameters such as inspiratory time (Ti), expiratory time (Te), total breath timing (Ttot), peak inspiratory flow (PIF), tidal volume (Vt) and minute ventilation (Vi) were then calculated from the breath timing data. The algorithm data for each of these parameters was then compared for the synthetic and clinical datasets.

Bias and variability of these differences are reported. Bland-Altman tests and linear regression were undertaken in MATLAB to assess the performance of the algorithm.

## Results

### Synthetic dataset

#### Effect of sampling frequency and noise on the algorithm

**Fig 5:**
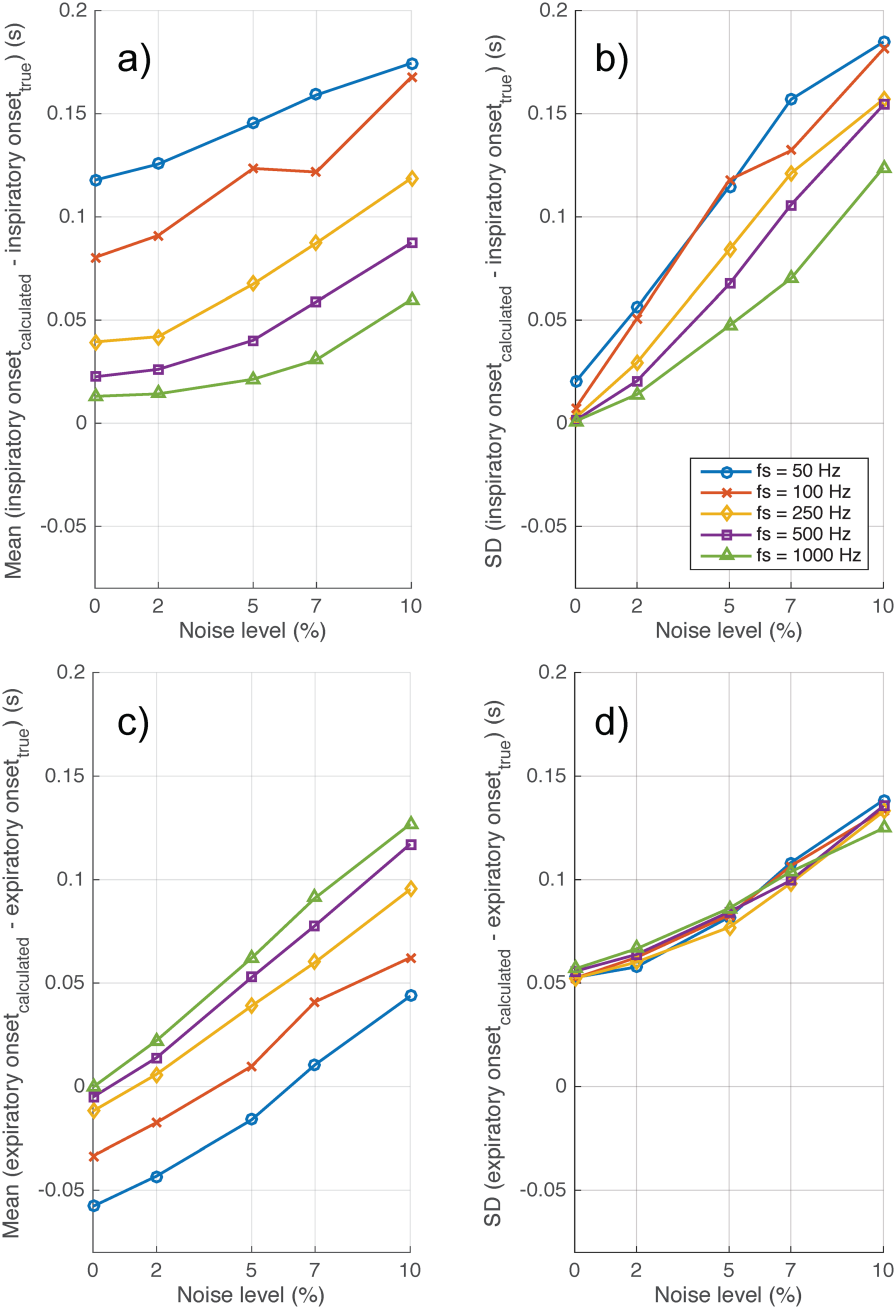
Effect of sampling frequency and random noise on the accuracy of the proposed algorithm to detect inspiratory (a-b) and expiratory onsets (c-d). Noise level of less than 5% in the flow signal is typical to that seen in most experimental settings.

**Fig 6:**
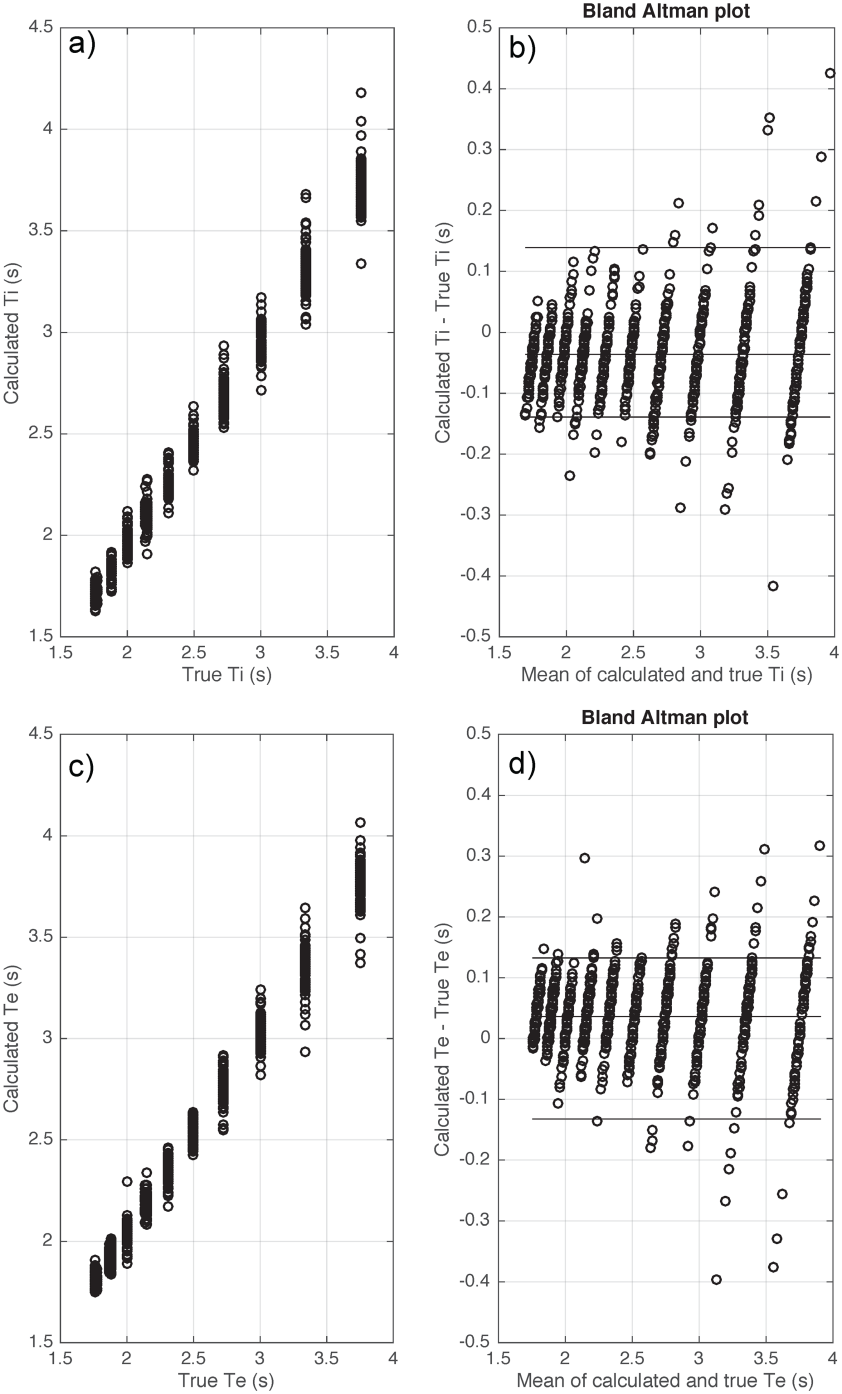
Bias and variability of inspiratory time (Ti) (a-b) and expiratory time (Te) (c-d) when applying the proposed algorithm on synthetic data of 1000 breaths generated by a mathematical model with a sampling frequency fs = 250 Hz and noise level = 2%. True Ti and Te are the reference inspiratory and respiratory times from the noise-free flow signal generated by our mathematical model. Calculated Ti and Te are the calculated inspiratory and respiratory times from the noisy signal (after different types of noise were added to disturb the baseline), generated by our model.

Fig 5 shows the effect of sampling frequency and noise on bias and variability of the algorithm. Both bias and variability increased with increased noise, while increased sampling frequency reduced both bias and variability. Given the noise level in a typical flow signal is less than 5% (refer to Figure B in S1 File) and sampling frequency is greater or equal to 250 Hz (from our laboratory), the bias and variability are less than 0.1 (s). Fig 6 shows the bias and variability of Ti and Te from a synthetic signal of 1000 breaths generated by a mathematical model with sampling frequency = 250 Hz and noise level = 2%.

### Bias and variability of fundamental respiratory parameters

**Fig 7:**
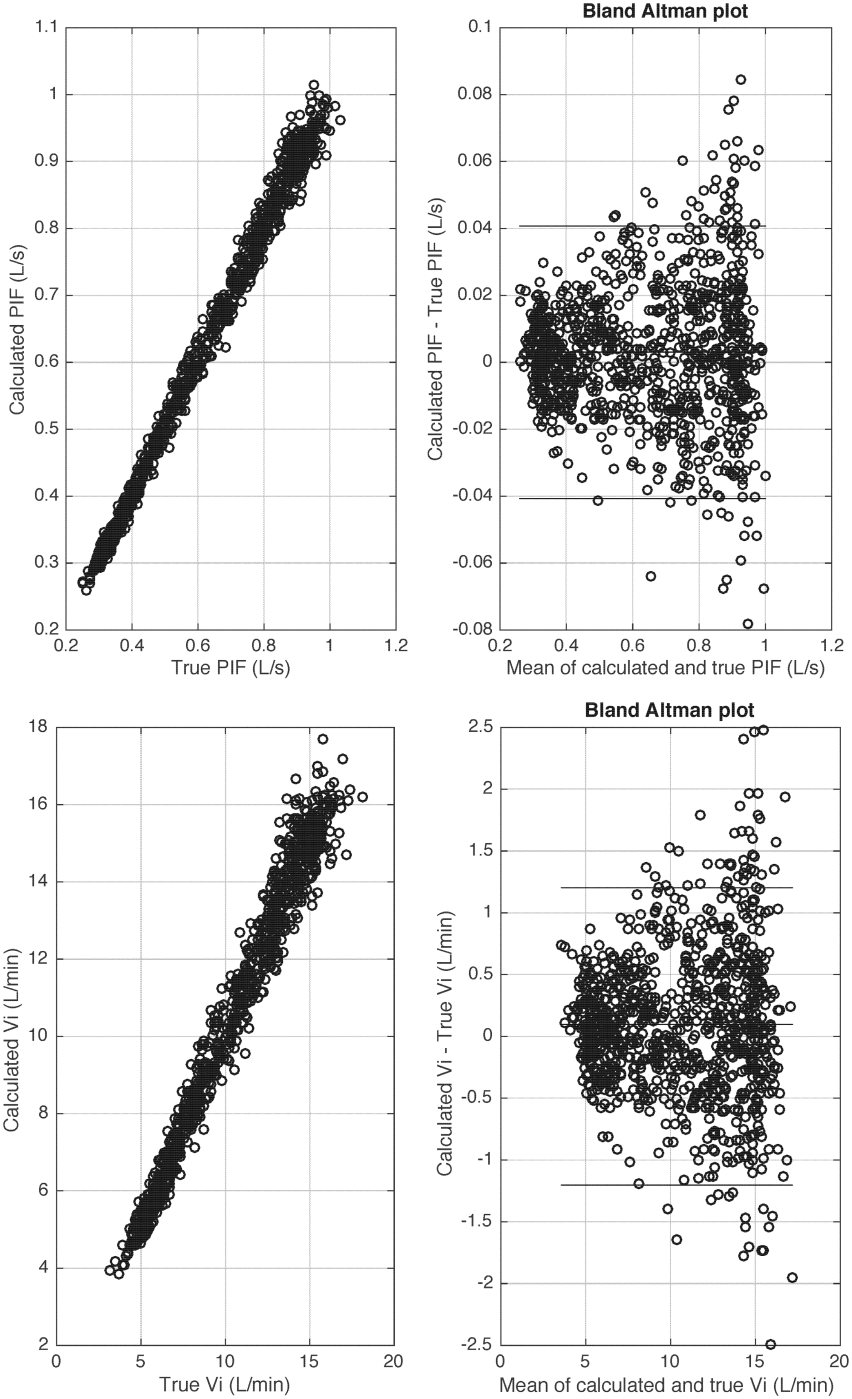
Bias and variability of peak inspiratory flow (PIF) (a-b) and minute ventilation (Vi) (c-d) when applying the proposed algorithm on synthetic data of 1000 breaths generated by a mathematical model with sampling frequency fs = 250 Hz and noise level = 2%. True PIF and Vi are the reference PIF and Vi calculated from the noise-free flow signal, generated by our mathematical model. Calculated PIF and Vi are the calculated PIF and Vi from the noisy signal (after different types of noise were added to disturb the baseline), generated by our model.

In Fig 7 and Table 1, we report the results of the model with a sampling frequency = 250Hz and noise level = 2% as these parameters reflect typical respiratory signals from our laboratory. Fig 7 shows bias and variability of PIF and Vi. Table 1 shows errors of key respiratory parameters. R^2^ values between the calculated and true respiratory parameters are all > 0.97.

**Table 1:** Errors of calculated respiratory parameters calculated from synthetic data of 1000 breaths generated by a mathematical model with sampling frequency = 250 Hz and noise level = 2%.

	Absolute Error	Relative Error (%)	R^2^	SSE
**Ti** (s)	-0.036 ± 0.069	-1.544 ± 2.572	0.988	0.068
**Te** (s)	0.036 ± 0.066	1.555 ± 2.451	0.989	0.066
**Ttot** (s)	0.0001 ± 0.042	0.006 ± 0.851	0.999	0.042
**PIF** (L/s)	0.003 ± 0.020	0.556 ± 3.056	0.992	0.020
**Vt** (L)	0.009 ± 0.053	1.211 ± 5.686	0.982	0.052
**Vi** (L/min)	0.097 ± 0.602	1.206 ± 5.620	0.974	0.599

Ti: inspiratory time; Te: expiratory time; Ttot: total duration of a breath; PIF: peak inspiratory flow; Vt: tidal volume; Vi: minute ventilation; SSE: sum of squared errors. R^2^ and SSE were calculated from linear regression analysis. Data are presented as mean ± SD.

### Clinical dataset

#### Participant characteristics for the clinical dataset

Data were extracted from 6 people with OSA and 4 healthy controls of similar age and BMI range (Table 2). In total, 20 minutes of data during wakefulness, 40 minute of sleep data on therapeutic CPAP (or 4 – 5cmH_2_O in the healthy controls) immediately preceding each CPAP reduction and 40 minutes of data during transient CPAP reductions during NREM sleep were extracted for analysis.

**Table 2:** Participant characteristics.

	Controls	OSA
**# Subjects**	4	6
**Age** (years)	37 ± 19	46 ± 15
**Sex**	2M / 2F	6M / 0F
**BMI** (kg/m^2^)	25 ± 3	27 ± 5
**AHI** (# events/h sleep)	1 ± 2	35 ± 14
**# Analyzed breaths during wakefulness**	152	181
**# Analyzed breaths during sleep**	464	632
**Pmask** (cmH_2_O)		
**Pre- CPAP reduction**	5.6 ± 1.0	9.0 ± 3.7
**During CPAP reduction**	-2.2 ± 2.8	2.8 ± 2.4
**Difference**	7.7 ± 3.2	6.2 ± 2.6

BMI: Body mass index; AHI: apnea hypopnea index; Pmask: mask pressure; CPAP: continuous positive airway pressure; Data are presented as mean ± SD.

### Performance of the algorithm in the clinical dataset

#### Using the flow signal

Table 3 shows the performance of the proposed algorithm in detecting inspiratory and expiratory onsets from the clinical dataset. Using the flow signal, the inspiratory/expiratory onsets have bias ≤ 0.023 (s) and variability ≤ 0.089 (s), and only 2.4% (8 breaths) of the total number of analyzed breaths (N = 333) were missed during wakefulness. Similarly, the algorithm could detect the inspiratory/expiratory onsets with bias ≤ 0.01 (s) and variability ≤ 0.08 (s) and missed 5% (28 breaths) of the total number of analyzed breaths (N = 562) during sleep.

**Table 3:** Performance of the proposed algorithm (compared to visual expert analysis) in detecting inspiratory and expiratory onsets from flow signal and epiglottic pressure signal (Pepi).

	Flow signal		Pepi signal	
	Wakefulness	Sleep	Wakefulness	Sleep
**Number of breaths analyzed**	333	562	N/A	534
**Missed breaths** (%)	2.4	4.9	N/A	11.6
**Difference in inspiratory onsets** (mean ± SD) (s)	0.023 ± 0.089	-0.010 ± 0.074	N/A	-0.031 ± 0.156
**Difference in expiratory onsets** (mean ± SD) (s)	-0.006 ± 0.056	-0.003 ± 0.028	N/A	-0.009 ± 0.147

Difference in inspiratory onset = calculated inspiratory onset—expert detected inspiratory onset

Difference in expiratory onset = calculated expiratory onset—expert detected expiratory onset

Data are presented as mean ± SD.

**Fig 8:**
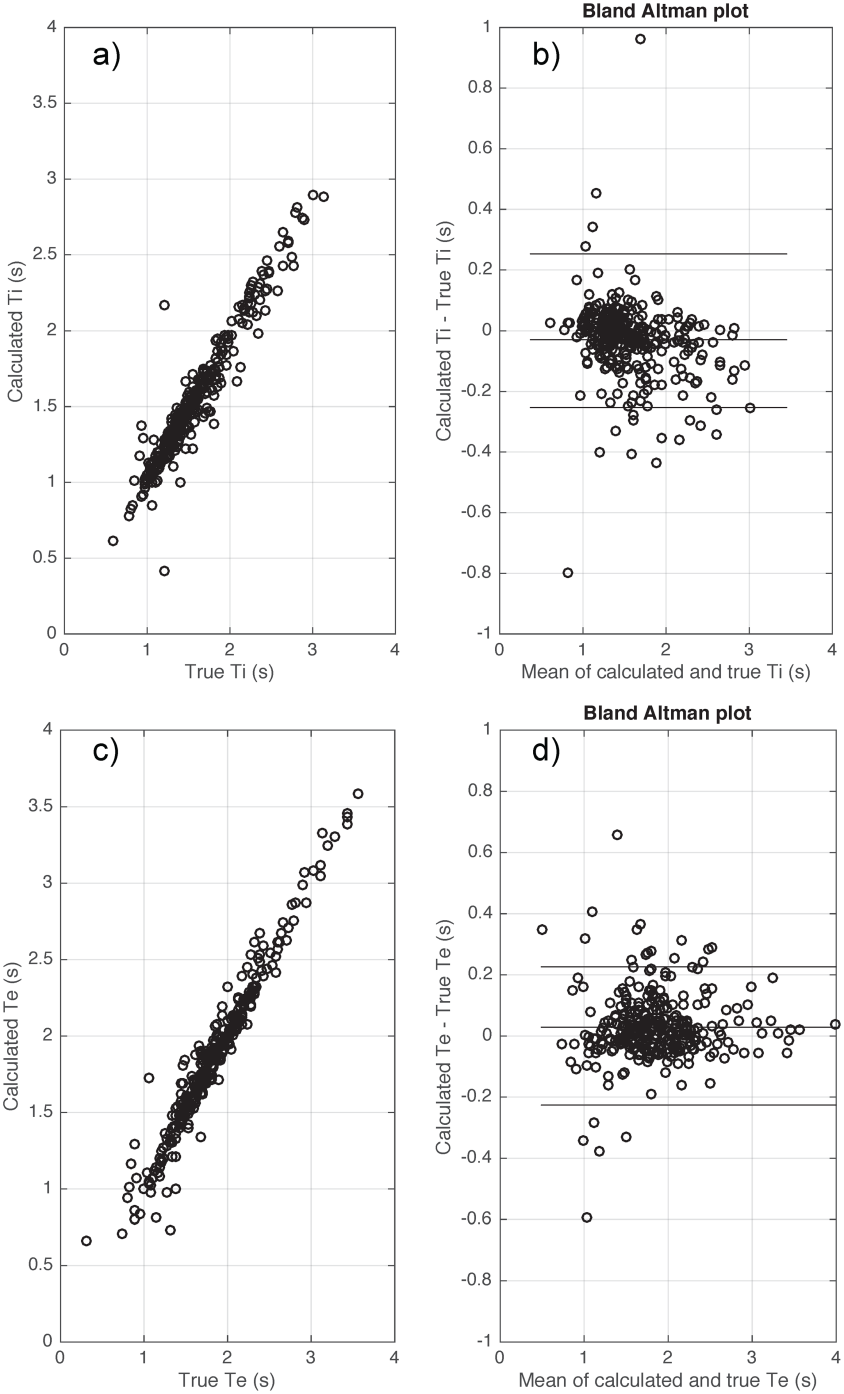
Bias and variability of inspiratory time (Ti) (a-b) and expiratory time (Te) (c-d) when applying the proposed algorithm on flow signals of clinical datasets during transient reductions in continuous positive airway pressure during wakefulness. True values were visually scored by an expert investigator blinded to the algorithm data.

Table 4 shows the errors for each calculated respiratory parameter from the clinical dataset compared to expert detected values. Using the flow signal, the calculated breath timing (Ti, Te and Ttot) had a small absolute error with bias ≤ 0.029 (s) and variability ≤ 0.126 (s). R^2^ values between the calculated and true breath timing parameters were all > 0.92. Fig 8 shows the Bland Altman plot of the calculated breath timing versus true breath timing using the flow signal during wakefulness.

**Table 4:** Errors of calculated respiratory parameters calculated from flow signal of the clinical datasets during wakefulness.

	Absolute Error	Relative Error (%)	R^2^	SSE
**Flow signal** (N = 333 breaths)				
**Ti** (s)	-0.029 ± 0.126	-1.141 ± 9.176	0.922	0.117
**Te** (s)	0.029 ± 0.113	2.043 ± 10.136	0.955	0.113
**Ttot** (s)	0.0001 ± 0.108	0.127 ± 4.192	0.984	0.107
**PIF** (L/s)	-0.009 ± 0.089	0.121 ± 13.819	0.914	0.084
**Vt** (L)	-0.026 ± 0.149	0.853 ± 22.083	0.922	0.129
**Vi** (L/min)	-0.340 ± 2.535	0.073 ± 19.615	0.858	2.336
**Nadir Pepi** (cmH_2_O)	0.043 ± 0.682	1.797 ± 19.269	0.938	0.640

Ti: inspiratory time; Te: expiratory time; Ttot: total duration of a breath; PIF: peak inspiratory flow; Vt: tidal volume; Vi: minute ventilation; SSE: sum of squared errors. R^2^ and SSE were calculated from linear regression analysis. Data are presented as mean ± SD.

**Fig 9:**
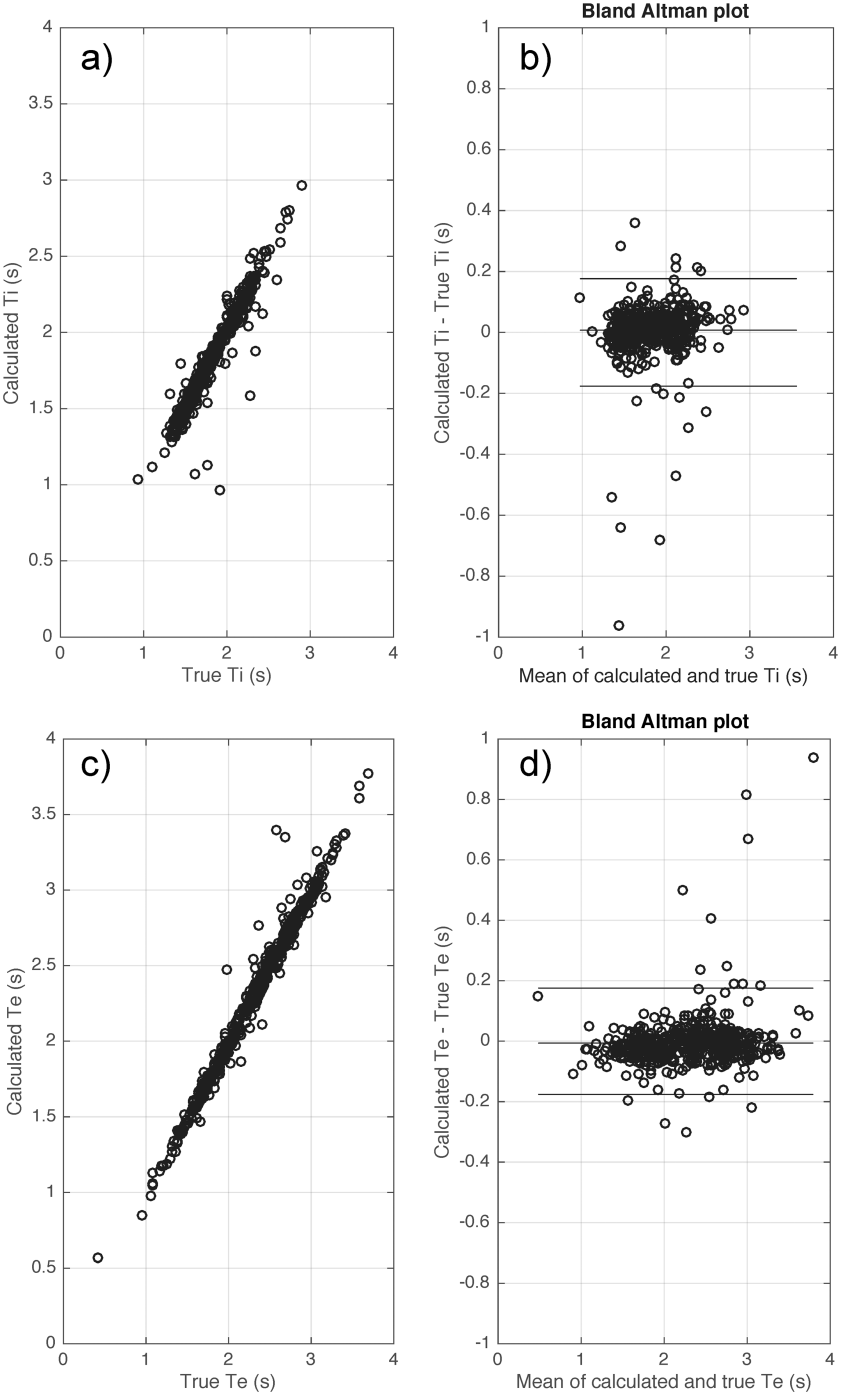
Bias and variability of inspiratory time (Ti) (a-b) and expiratory time (Te) (c-d) when applying the proposed algorithm on the flow signals of clinical datasets during transient reductions in continuous positive airway pressure during sleep. True values were visually scored by an expert blinded to the algorithm data.

Table 5 shows the errors for each calculated respiratory parameter from the clinical dataset compared to expert detected values during sleep. The absolute error was small with bias ≤ 0.01 (s) and variability ≤ 0.11 (s) when using the flow signal to calculate breath timing (Ti, Te and Ttot). R^2^ values between the calculated and true breath timing parameters were all >0.9. Fig 9 shows the Bland Altman plot of the calculated breath timing versus true breath timing using the flow signal during sleep.

**Table 5:** Errors of calculated respiratory parameters calculated from flow and epiglottic pressure signal of clinical datasets during sleep on CPAP.

	Absolute Error	Relative Error (%)	R^2^	SSE
**Flow signal** (N = 562 breaths)				
**Ti** (s)	-0.007 ± 0.088	-0.465 ± 4.748	0.919	0.088
**Te** (s)	-0.006 ± 0.088	-0.363 ± 3.909	0.974	0.088
**Ttot** (s)	0.0001 ± 0.101	0.014 ± 2.361	0.977	0.101
**PIF** (L/s)	0.009 ± 0.052	2.283 ± 10.371	0.939	0.052
**Vt** (L)	0.015 ± 0.085	3.523 ± 13.455	0.874	0.084
**Vi** (L/min)	0.212 ± 1.387	3.069 ± 13.903	0.882	1.370
**Nadir Pepi** (cmH_2_O)	-0.089 ± 0.477	4.818 ± 20.078	0.984	0.478
**Pepi signal (N = 534 breaths)**				
**Ti** (s)	0.022 ± 0.211	1.629 ± 11.676	0.797	0.207
**Te** (s)	-0.016 ± 0.222	-0.267 ± 11.291	0.883	0.220
**Ttot** (s)	0.007 ± 0.212	0.237 ± 5.128	0.899	0.212
**PIF** (L/s)	0.007 ± 0.111	3.265 ± 29.651	0.889	0.111
**Vt** (L)	0.004 ± 0.186	3.333 ± 34.145	0.663	0.181
**Vi** (L/min)	0.041 ± 2.710	3.687 ± 52.788	0.774	2.701
**Nadir Pepi** (cmH_2_O)	-0.253 ± 0.969	9.375 ± 18.245	0.974	0.965

Ti: inspiratory time; Te: expiratory time; Ttot: total duration of a breath; PIF: peak inspiratory flow; Vt: tidal volume; Vi: minute ventilation; Nadir Pepi: Nadir epiglottic pressure. SSE: sum of squared errors. R^2^ and SSE were calculated from linear regression analysis. Data are presented as mean ± SD.

#### Using epiglottic pressure signal (Pepi)

Table 3 shows the performance of the proposed algorithm in detecting inspiratory and expiratory onsets using the Pepi signal from the clinical dataset during sleep. Specifically, the algorithm detected the inspiratory/expiratory onsets with bias ≤ 0.031 (s) and variability < 0.16 (s), and missed 11.6% (62 breaths) of the total number of breaths detected manually (N = 534).

**Fig 10:**
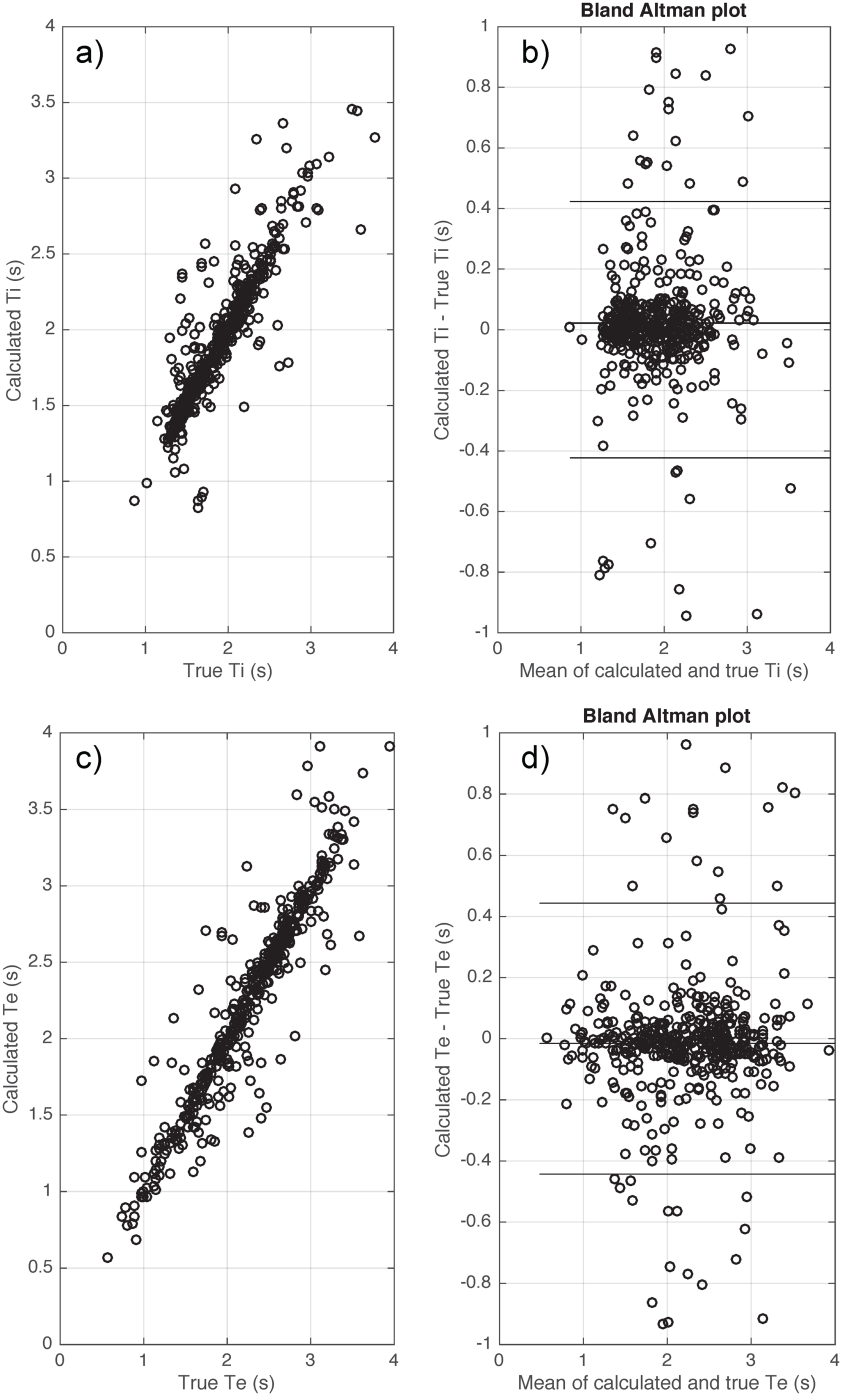
Bias and variability of inspiratory time (Ti) (a-b) and expiratory time (Te) (c-d) when applying the proposed algorithm on the epiglottic pressure signals of clinical datasets during transient reductions in continuous positive airway pressure during sleep. True values were visually scored by an expert investigator blinded to the algorithm data.

Table 5 shows the errors of all of the calculated respiratory parameters from the clinical dataset compared to expert detected values during sleep. Using the Pepi signal, the calculated breath timing (Ti, Te and Ttot) had an absolute error with bias ≤ 0.033 (s) and variability ≤ 0.2 (s). R^2^ values between the calculated and true breath timing are all > 0.79. Fig 10 shows the Bland Altman plot of the calculated breath timing versus true breath timing using the Pepi signal during sleep.

## Discussion

In this paper, we present a novel signal processing method for breath detection during variable mask pressures in awake and sleeping humans. One of the major advantages of our algorithm is that it can be applied on both flow and pressure signals, enabling automated breath detection when one of the signals are not available due to technical artifacts and/or physiological events such as obstructive apneas. This feature of the algorithm has important implications, including facilitation of sleep apnea phenotyping approaches that require accurate breath detection in large data sets, as well as multiple other research applications in which time-consuming, labor-intensive, manual breath detection processes have traditionally been required. We have also validated the algorithm under a variety of conditions to examine the effect of different types of noise, sampling frequencies and variable mask pressures on its performance.

When applied to the flow signal, our algorithm is very accurate in detecting the onsets of inspiration and expiration during both wakefulness and sleep and during variable mask pressures. The results from our mathematical model indicate that higher sampling frequencies of the flow signal improve the noise tolerance of the algorithm. The findings suggest that sampling frequencies of equal to or greater than 250Hz are desirable for most research settings in order to achieve accurate breath detection. In the clinical dataset, the algorithm correctly identified approximately 98% of the analyzed breaths and detected inspiratory and expiratory onsets with minimal difference (≤23 ms) compared to expert visual detection during both wakefulness and during sleep with variable mask pressures. This is comparable to the results of the most accurate previously published algorithm which detected a mean difference in the onset of inspiration of 34 ± 71 ms and 5 ± 46 ms for expiration with 98% of the breaths being correctly identified [9]. However, our dataset contained much larger levels of baseline drift introduced by variable mask pressures compared to previous work. Furthermore, the proposed algorithm is simple to implement and operate, and does not require training datasets (with expert scored data) compared to the previous one [9]. Unlike traditional breath detection algorithms [7–9] that define the onsets of inspiration/expiration as the points where the flow signal crosses zero, our algorithm defines the “true” onset of inspiration as the points at which inspiratory effort commences as reflected by a sharp change in the shape of the flow signal—an inflection point. This, therefore, can overcome the problematic volume drift correction during conditions where mask pressure is variable or breathing is unstable [8].

Detecting breaths from the epiglottic pressure signal is more challenging compared to flow due to higher level of noise and nonlinear variation of the baseline. This is the first study that we are aware of that has attempted to automatically detect breaths using an epiglottic pressure signal which has traditionally been reserved for time-consuming manual approaches. Our algorithm correctly identified 89% of breaths and detected inspiratory and expiratory onsets with a mean difference ≤ 31 ms with variable mask pressures during sleep. Although accuracy was reduced when using pressure compared to flow, the performance of our algorithm on the pressure signal is still indistinguishable to a previously reported study on inter-expert variability, whereby five experts scored breaths using flow signals with 95% confidence intervals of 220.5 ms and 100.6 ms for inspiration and expiration, respectively [9].

Some limitations need to be noted. Firstly, the use of the inflection point will be problematic under certain conditions. For example in severe chronic obstructive pulmonary disease (COPD) the flow and pressure inflection points will not be aligned in time due to the presence of inspiratory PEEP (positive end expiratory pressure). However, this is also an issue for other breath detection approaches such as zero crossing. Secondly, the algorithm relies on recognition of the inflection points to detect breaths. Hence, high signal-to-noise ratio is required for the algorithm to operate correctly. Most of the breaths that failed to be detected in this study were due to signal quality where it is difficult to detect inflection points in the signal, even with a careful manual approach. Therefore, it should be noted that the proposed algorithm still requires expert oversight for correction of occasional errors in breath detection where signal quality is poor. Nonetheless, this is only a fraction of the time that would be required to complete the entire task manually and arguably may be more accurate by avoiding potential human error for the majority of the analysis tasks. The number of breaths used for validation was also relatively small (~1000). Finally, this study has scored data from only one expert and there can be inter-expert bias with manual scoring. This potential bias is another reason for our desire to develop an automated approach. While including an extra scorer would provide a measure of the degree of inter-expert bias, this was not the goal of the current study. Rather, we believe that our dual approach of assessing the performance of the algorithm both with the model simulation and an experienced scorer (current gold standard accepted in the published literature) is appropriate.

In terms of future directions, it will be important to assess the performance of the algorithm in different datasets. There is also potential to use the algorithm across a range of clinical settings. For example, it could be used to gain an estimate of overnight mean and peak airflow using standard airflow signals acquired during polysomnography similar to recent techniques that have been proposed as simplified approaches to estimate airway collapsibility [24] to facilitate treatment decisions for sleep disordered breathing.

In conclusion, we have developed and validated a new breath detection algorithm based on the inflection points of flow or epiglottic pressure signal. The algorithm has excellent performance and is robust to baseline drifts and noise during variable mask pressures. This algorithm has major implications in terms of reducing the highly time-consuming, labor-intensive burden that has traditionally been required for accurate breath detection for sleep apnea phenotyping techniques and respiratory physiology research.

## Supporting information

S1 File Synthetic signal from mathematical model. (PDF)

S2 File Breath detection MATLAB source codes. (ZIP)
