# Supplementary material for: An automated and reliable method for breath detection during variable mask pressures in awake and sleeping humans
Source: PLoS One. 2017 Jun 13;12(6):e0179030. doi: 10.1371/journal.pone.0179030 (PMC5469467; doi:10.1371/journal.pone.0179030)
Supplement: S1 File — (PDF) [file pone.0179030.s001.pdf]

Supporting information

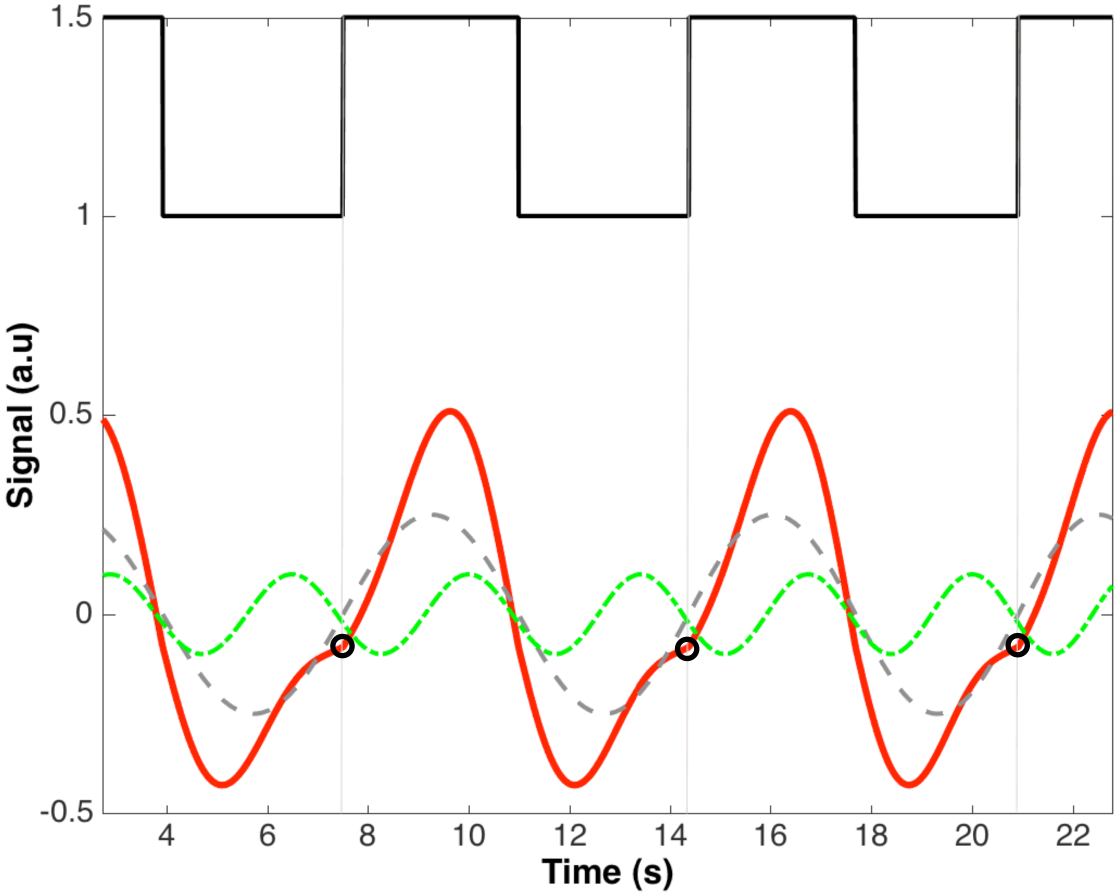

Figure A. Flow waveform (solid red line) with inflection points (circles) generated by a mathematical model of a sine waveform (dash grey line), a cosine waveform (dash green line), and a square waveform (solid black line). The positions of inflection points were set by the square waveform.

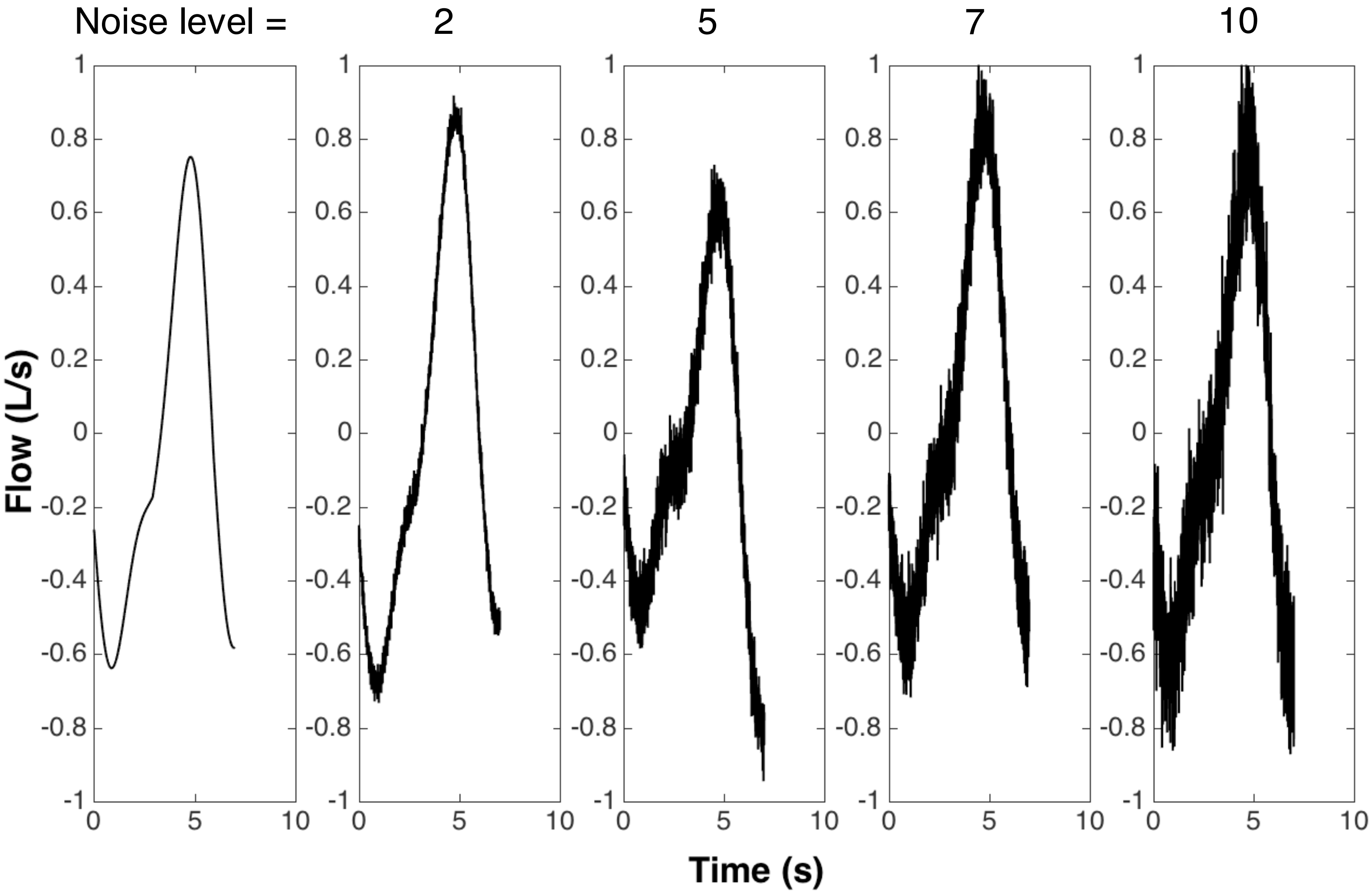

Figure B. Simulated flow signal with different noise level. Typical measured flow signal from our laboratory is often less than 5%.
